# Identification of immune-related target and prognostic biomarkers in PBMC of hepatocellular carcinoma

**DOI:** 10.1186/s12876-023-02843-y

**Published:** 2023-07-12

**Authors:** Rui Hu, Wei Zhang, Zhiyi Han, Mengqing Ma, Qi Huang, Minling Lv, Wenfeng Ma, Xinfeng Sun, Wenxing Feng, Jing Li, Xin Zhong, Jialing Sun, Wei Yao, Xiaozhou Zhou

**Affiliations:** 1 Department of Liver Disease, Shenzhen Traditional Chinese Medicine Hospital, Futian District, Shenzhen, 518033 Guangdong Province China; 2 Department of Liver Disease, The Fourth Clinical Medical College of Guangzhou University of Chinese Medicine, Shenzhen, 518033 China

**Keywords:** Biological techniques, Hepatocellular carcinoma, CLDN18, Prognosis, Immune infiltration, T cells

## Abstract

**Background:**

Hepatocellular carcinoma (HCC) is the third leading cause of cancer-related deaths worldwide, and is characterized by insidious onset, rapid progression, and poor prognosis. Immunotherapy is a first-line treatment for advanced HCC. The identification of immune-related prognostic markers may be an effective strategy to predict and improve clinical response rate of immunotherapy.

**Methods:**

The DESeq2, edgeR, and limma R packages were used to compare the transcriptomes of HCC with different prognoses. Cancer-related databases such as UALCAN, TNMplot, GEPIA, muttarget and Human Protein Atlas (HPA), and the Kaplan–Meier Plotter platform were used to analyze the relationship between CLDN18 and the clinical characteristics, as well as prognosis of HCC. The co-expressed genes of CLDN18 were obtained from LinkedOmics platform, and GO functional enrichment and KEGG pathway analysis were performed. The CIBERSORT, TIMER, Timer 2.0 and TISIDB algorithms were used to analyze immune infiltration.

**Results:**

CLDN18 was differentially expressed in HCC patients with different prognoses, and its expression level in PBMC was positively correlated with the stage of BCLC. In addition, CLDN18 was significantly overexpressed in HCC tumor tissues compared to adjacent non-tumor tissues, which was consistent with PBMC sequencing results and immunohistochemical data from human protein profiles. CLDN18 was also positively correlated with HCC staging and grading, and high expression levels of CLDN18 predicted shorter overall survival. Functional annotation of CLDN18 in HCC revealed enrichment of the cellular senescence and protein activation cascade, along with biological processes such as cell cycle, inflammatory response, and cellular ketone metabolism. In addition, CLDN18 was also associated with tumor infiltrating immune cells, suppressive immune cell markers, T lymphocyte depletion and activation of HCC, and low expression of CLDN18 was associated with higher CD8 + T cell infiltration and better survival rates.

**Conclusions:**

CLDN18 is a potential prognostic marker and immunotherapeutic target for HCC.

**Supplementary Information:**

The online version contains supplementary material available at 10.1186/s12876-023-02843-y.

## Introduction

Hepatocellular carcinoma (HCC) accounts for 75%-85% of primary liver cancer cases. According to the Global Cancer Report, approximately 830,000 people died of liver cancer in 2020, accounting for 8.3% of all cancer-related deaths, thereby making it the third leading cause of cancer-related deaths after lung cancer (18%) and colorectal cancer (9.4%) [1]. Chronic infection with hepatitis B virus (HBV) and hepatitis C virus (HCV), alcohol abuse, smoking, long-term exposure to fungal metabolites, and metabolic diseases such as non-alcoholic fatty liver disease and diabetes are common risk factors of HCC. The 5-year survival rate of early-stage HCC patients after surgical resection or radiofrequency ablation can be as high as 50%-70% [2, 3]. However, most patients are in the advanced stage of the disease when first diagnosed, of which only 30%-40% can undergo curative surgery [4]. Given that immune escape is one of the key features of HCC, immunotherapies such as immune checkpoint inhibitors (ICIs), adoptive cell transfer, tumor vaccines etc, have been the focus of advanced HCC treatment in recent years [5]. The 2020 ASCO guidelines recommend atezolizumab combined with bevacizumab as the first-line therapy for most cases of advanced HCC [6]. Unfortunately, not all patients benefit from immunotherapy given the heterogeneity in the number, function, and spatial organization of the tumor-infiltrating immune cells [7, 8]. In addition, the clonal expansion and cytotoxicity of T cells are key factors affecting the response to immune checkpoint blockade [9]. There is an urgent need to explore novel prognostic biomarkers for developing individualized immunotherapy regimens against HCC.

Although several diagnostic biomarkers have been identified for the early screening of HCC [10, 11], the molecular mechanisms underlying tumor progression and disease prognosis remain to be elucidated. The transcriptome of peripheral blood mononuclear cells (PBMCs) is of a single origin and can reflect the local immune cell invasion in the tumor microenvironment (TME) [12]. Furthermore, blood samples are easier to retrieve compared to tumor tissues, and blood tests are less invasive, and have higher patient cooperation and reproducibility. The aim of this study was to explore biomarkers related to immune invasion and prognosis by comparing the transcriptomes of PBMCs isolated from HCC patients with different prognostic outcomes.

## Materials and methods

### Sample collection

Twelve HCC patients with BCLC stages C-D were recruited, and 8 mL peripheral blood was drawn from each patient using EDTA anticoagulant vacuum angiography. PBMCs were isolated from whole blood by density gradient centrifugation, and RNA was extracted from the cells using TRIzol reagent according to the manufacturer’s instructions. The RNA samples are stored at -80℃, and subsequently sequenced as per established protocols. The enrolled patients were followed up regularly. All experiments were performed in accordance with the relevant regulations of the Ethics Committee, and the study was approved by the Ethics Committee of Shenzhen Traditional Chinese Medicine Hospital.

### RNA-sequencing library

The RNA samples were first enriched for mRNA by removing rRNA according to the Illumina TruSeq RNA sample preparation guidelines (Illumina, San Diego, California, USA), and then reverse transcribed to cDNA. The sequencing connector was added to the double stranded DNA. The library was tested for quality and quantified using 2100 Bioanalyzer (Agilent). Double end sequencing (2 × 150 bp) was performed using Illumina HiSeq X TEN Reagent Kit (Illumina, San Diego, CA, USA).

### Bioinformatics analysis

The raw RNA-Seq data was filtered using SOAPnuke (version 1.0.1) and the high-quality and clean readings were aligned with the human transcriptome and genome HG19 using Tophat2 (version 2.0.7). All genes with a sample size of 0 were eliminated. R packages DESeq2, edgeR and limma were used to screen for the differentially expressed genes (DEGs). The UALCAN (http://ualcan.path.uab.edu) portal [13] and TNMplot (https://tnmplot.com/analysis/) [14] were used to analyze the differential expression levels of METTL7B, CLDN18, SOCS3, ITGA9 and RNASE3 in tumor and normal tissue samples in TGCA and MET500 datasets. The expression levels of CLDN18 proteins in HCC tissues were analyzed using the Human Protein Atlas database (https://www.proteinatlas.org/). Kaplan and Meier Plotter platform (http://kmplot.com/analysis/) was used to determine the prognostic relevance of CLDN18 in HCC in terms of the overall survival (OS) with data from GEO, EGA and TCGA. TIMER (https://cistrome.shinyapps.io/timer/) [15] and TIMER 2.0 (http://timer.cistrome.org/) [16] were used to analyze the correlation between CLDN18 and the infiltrating immune cell subtypes in HCC, and that between DEGs (CLDN18) and OS. The mutated genes affecting CLDN18 expression in HCC were identified using muTarget (http://www.mutarget.com/), an online platform of paired RNA-seq data and somatic gene mutations from 18 different cancer types. LinkedOmics database (http://www.linkedomics.org) [17] was used to conduct gene set enrichment analysis (GSEA) and KEGG pathway analysis for CLDN18.

### Statistical analysis

R (version 4.0.2) and GraphPad Prism 9 were used for statistical analysis. PCA and Pearson correlation analysis were performed to evaluate the similarity of samples. Spearman correlation analysis and the Kruskal–Wallis test were used to evaluate the association between CLDN18 and clinical characteristics of HCC. Log-rank test was used for Kaplan–Meier survival analysis. *P* < 0.05 was considered statistically significant.

## Results

### Differential gene expression of PBMC in different HCC subgroups

**Fig. 1:**
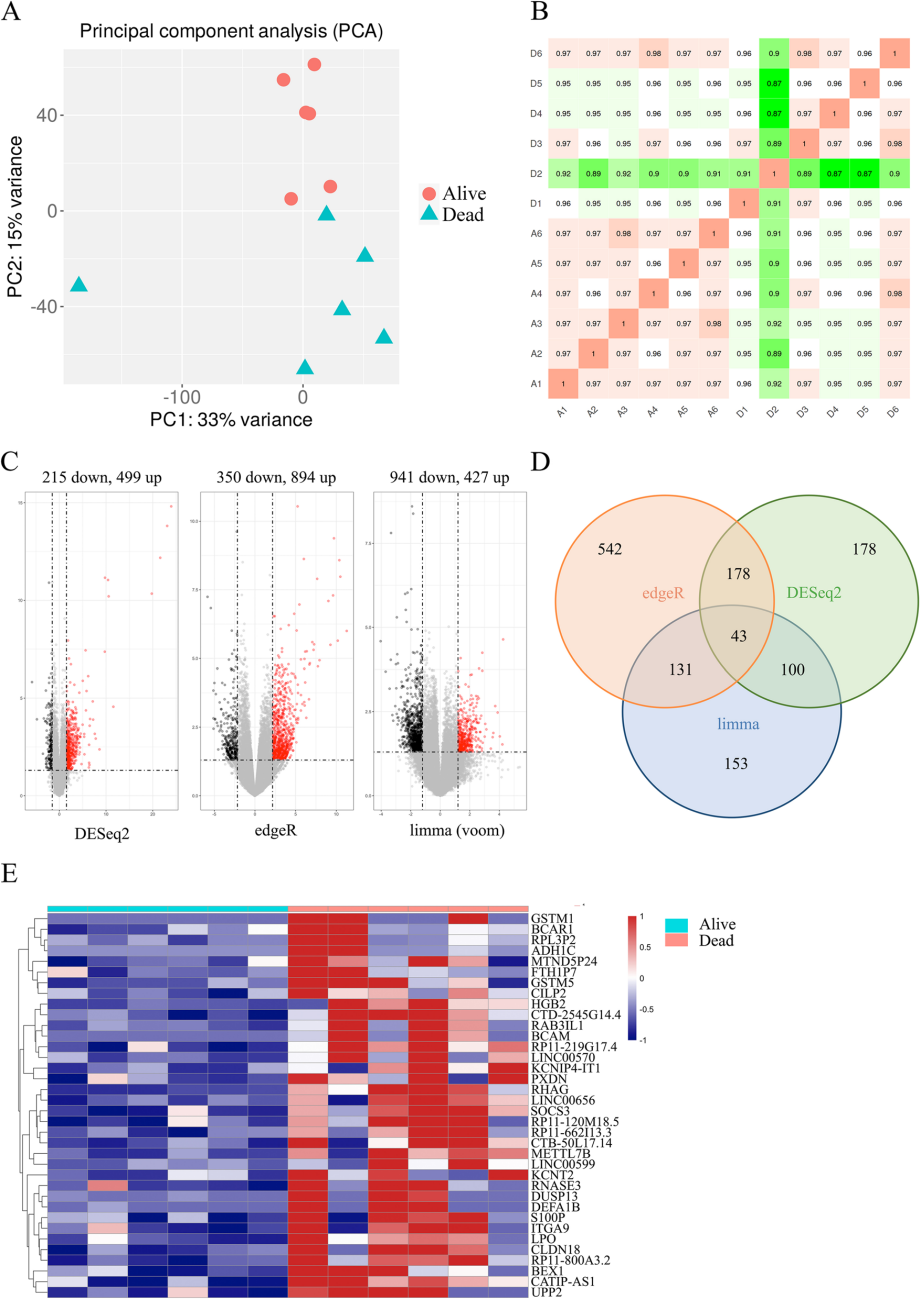
Identification of DEGs in the PBMCs of HCC subgroups. **A** Clustering diagram of PCA samples. **B** The heatmap based on Pearson correlation coefficient. **C** Volcano map showing DEGs obtained by DESeq2, edgeR and limma R package, *P* <0.05 and logFC cutoff of 95% confidence interval  **D** Venn plots showing intersecting DEGs between edgeR (orange circle), DESeq2 (green circle) and limma (orange circle). **E** The heatmap of the DEGs

Six of the enrolled patients had died by October 20, 2021. Principle component analysis (PCA) showed that the first principal component explained 33% of the overall difference, and the second explained 15% of the variance in the sample. Due to the basic transcriptomic characteristics of HCC, the survival and death patients were not significantly separated in PC1, but the genetic differences between the two groups were reflected in PC2. (Fig. 1A). Furthermore, the heatmap of sample correlation showed significant differences between the two groups, and the correlation within the HCC groups was significantly higher than that in the control group (Fig. 1B). The DEGs between the PBMC transcriptomes of the two patient groups were screened using *P* < 0.05 and logFC cutoff of 95% confidence interval as the criteria. The DESeq2, edgeR and limma R package revealed 499, 894 and 427 DEGs respectively (Fig. 1C), and Venn analysis showed that 43 DEGs were common to the three datasets (Fig. 1D). The DEGs were translated using genecode V22 official annotation file, and the heatmap is shown in Fig. 1E. The top 10 upregulated genes in the HCC group, including METTL7B, BCAM, CLDN18, DEFA1B, SOCS3, HBG2, GSTM1, RNASE3, ITGA9 and S100P, were selected for follow-up study.

### Identification of prognostically relevant genes in HCC

**Fig. 2:**
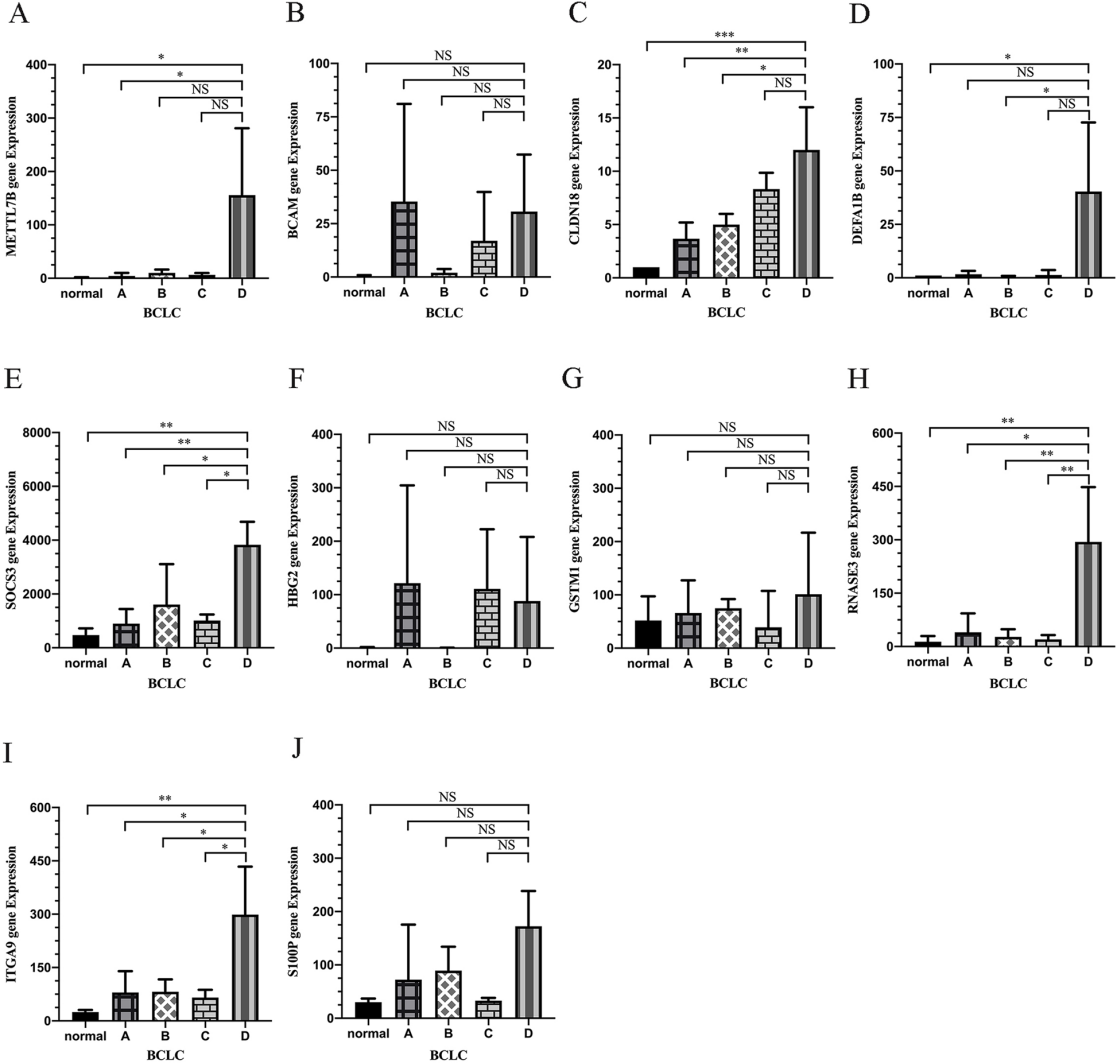
Identification of prognostically relevant genes in the PBMCs of HCC patients with different BCLC stages. The expression levels of METTL7B, BCAM, CLDN18, DEFA1B, SOCS3, HBG2, GSTM1, RNASE3, ITGA9 and S100P in the PBMCs of HCC patients with different BCLC stages. One-way ANOVA test was used for statistical analysis

To further confirm whether the above genes are associated with prognosis, we analyzed their expression in the PBMCs of 3 healthy patients and 12 HCC patients at BCLC stages A, B, C and D respectively. BACM, HBG2, GSTM1 and S100P showed similar expression levels across the different stages of HCC (Fig. 2B, F, G and J). DEFA1B was upregulated in stage D samples relative to normal tissues, whereas no significant difference (*P* = 0.0522) was observed for stages A and D. Since this does not meet the requirements of a predictive gene model, DEFA1B was excluded from subsequent analysis (Fig. 2D). In contrast, METTL7B, CLDN18, SOCS3, RANSE3 and ITGA9 were overexpressed in BCLC Stage D, and showed significant differences compared to each of the other stages, indicating a possible prognostic role in HCC (Fig. 2A, C, E, H and I). The immune landscape of HCC patients were further analyzed using the "CIBERSORT" algorithm. The results showed that monocytes are the main peripheral blood population in HCC patients, followed by resting NK cells. T cells, including CD8^+^T cells, naive CD4^+^T cells and resting memory CD4^+^T cells, formed the third largest subgroup in the PBMCs of HCC patients (Supplementary Fig. 1A and B). We eliminated subsets of immune cells with zero expression in most samples and analyzed the correlation between target genes and the remaining immune cells. METTL7B9 (COR = 0.467, *P* = 0.014), CLDN18 (COR = 0.390, *P* = 0.045), SOCS3 (COR = 0.796, *P* < 0.001), ITGA9 (COR = 0.582, *P* = 0.001) and RNASE3 (COR = 0.423, *P* = 0.028) were all positively correlated with monocytes and showed negative correlation with the CD8^+^T cells (Supplementary Fig. 1C).

### The expression of five candidate DEGs in HCC tissue samples

**Fig. 3:**
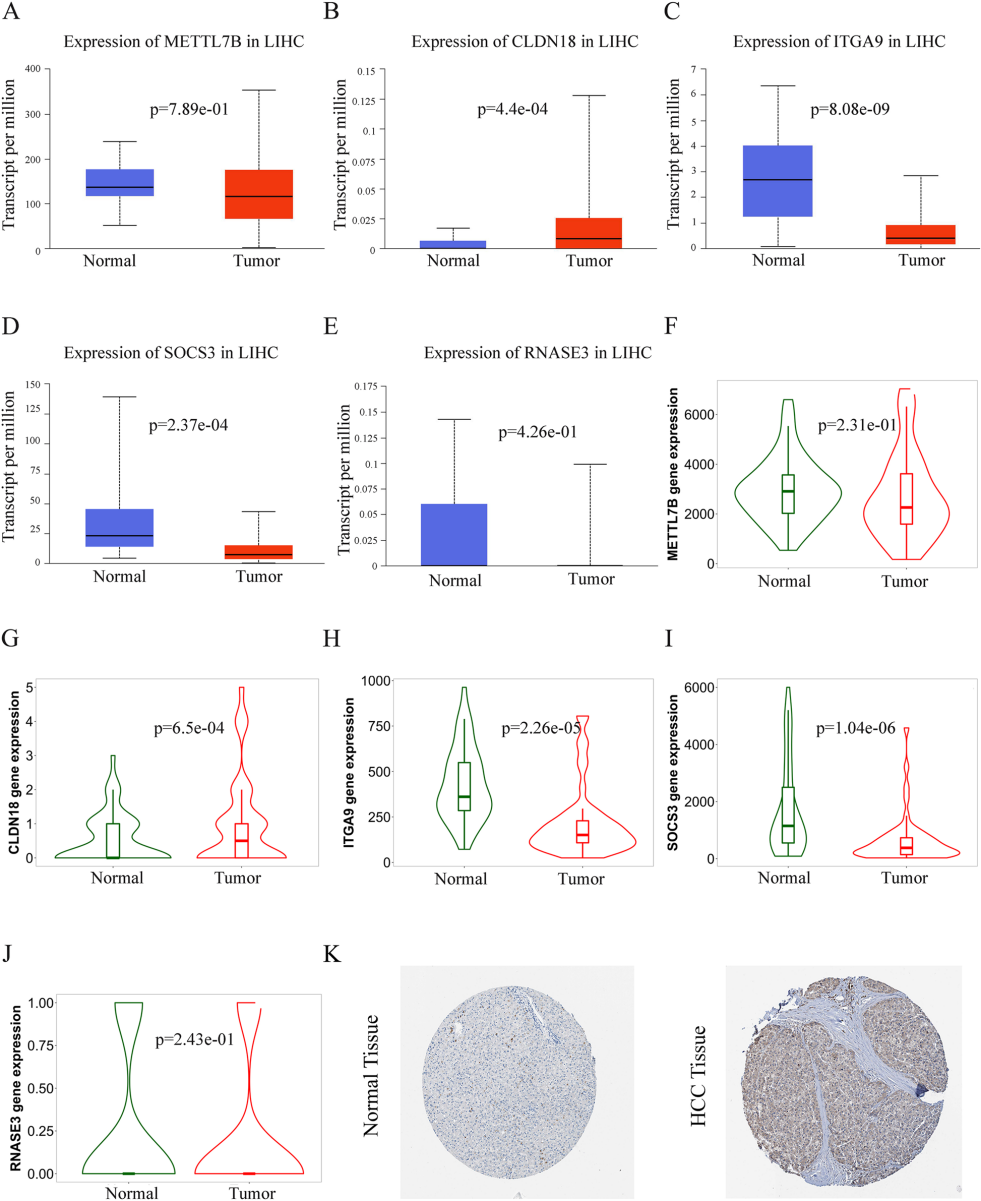
Expression levels of 5 candidate genes in HCC tissue samples. **A**-**E** Expression levels of METTL7B, CLDN18, SOCS3, ITGA9 and RNASE3 in normal liver tissues and 371 HCC samples using UALCAN. **F**-**J** METTL7B, CLDN18, SOCS3, ITGA9 and RNASE3 in normal tissues adjacent to HCC. **K** Immunohistochemistry IHC results of CLDN18 in HCC and normal tissues based on HPA

The five candidate genes identified above were then validated using the UALCAN platform based on the sequencing data of 371 HCC tumor tissues from TCGA database. The expression levels of CLDN18 (*P* = 4.4e-04) were significantly higher in HCC tissues compared to the normal tissues (Fig. 3B), whereas METTL7B, ITGA9, SOCS3 and RNASE3 were downregulated in the tumor tissues (Fig. 3A, C, D and E). Analysis of the TNMplot database indicated similar expression of METTL7B and RNASE3 (Fig. 3F and J), downregulation of ITGA9 and SOCS3 (Fig. 3H and I), and upregulation of CLDN18 (Fig. 3G) in the tumor tissues relative to the adjacent normal tissues. In conclusion, CLDN18 was upregulated in the tumor tissues as well as the PBMCs from HCC patients compared to the normal adjacent tissues and the PBMCs from healthy donors respectively. Furthermore, CLDN18 was highly expressed in the PBMCs of deceased BCLC stage C-D patients, and the expression levels were highest in patients with stage D. These results were validated by immunohistochemical data from human protein profiles (Fig. 3K).

### CLDN18 is associated with the clinical features of HCC

**Fig. 4:**
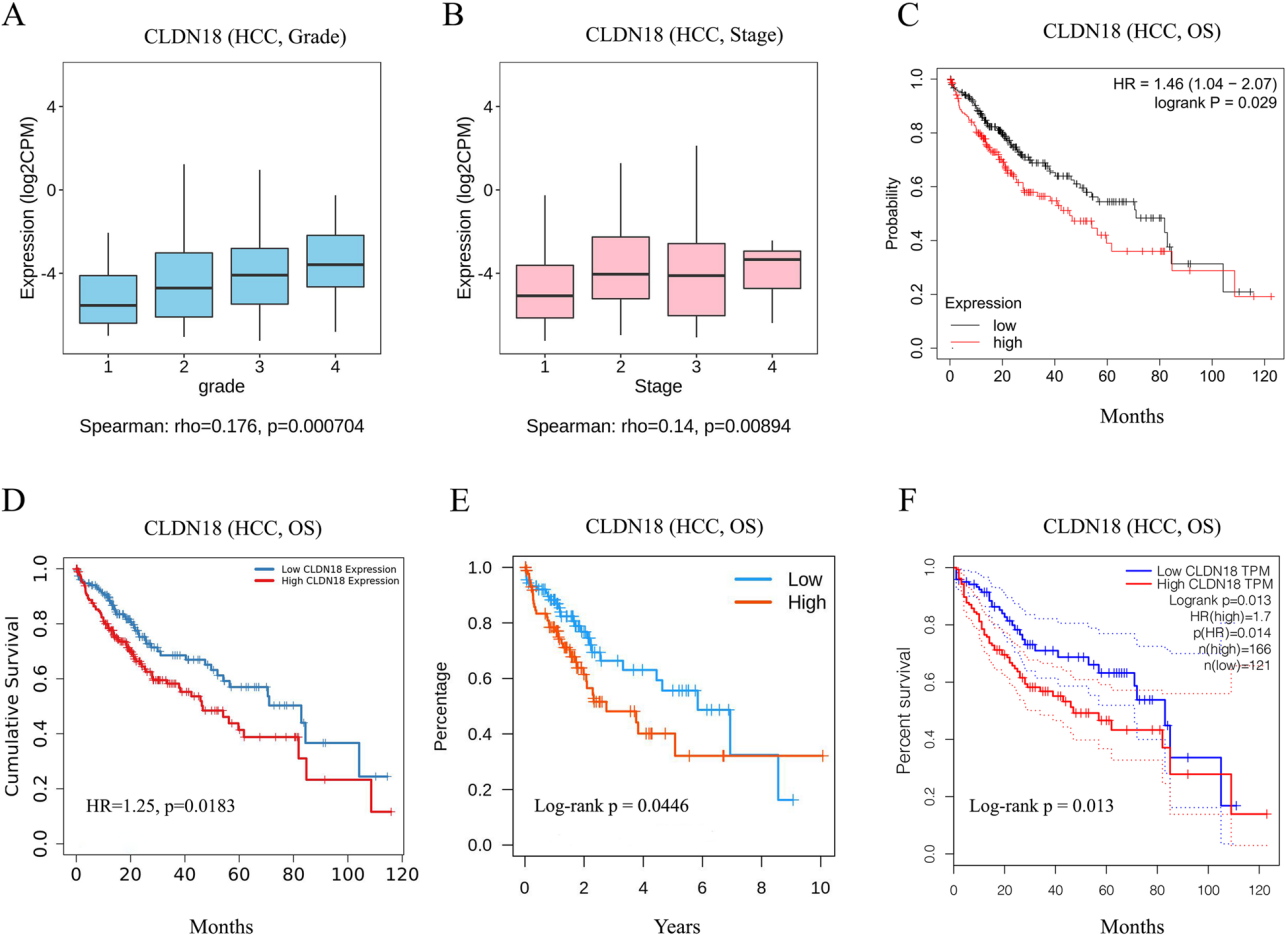
Correlation of CLDN18 expression with clinical characteristics and survival. **A**, **B** Correlation between CLDN18 and HCC stage and grade. **C**-**F** Relationship between CLDN18 and the overall survival of HCC patients (Kaplan–Meier Plotter, TIMER2.0, TISIDB and GEPIA)

We next analyzed the correlation between the levels of CLDN18 in HCC tissues and the clinicopathological features using the TISIDB database. CLDN18 (Spearman: rho = 0.176, *P* = 7.04E-04) were positively correlated with the HCC grade (Fig. 4A). In terms of tumor stage, CLDN18 expression levels increased with the increase in histological heterogeneity (Spearman: Rho = 0.14, *P* = 8.94E-03; Fig. 4B). Thus, the transcription level of CLDN18 in particular was prognostically significant. Survival analysis showed that high CLDN18 expression was associated with a shorter overall survival (OS: HR, 1.46, 95% CI from 1.04 to 2.07, log-rank *P* = 0.029; Fig. 4C), which was also verified in the TIMER2.0 (Fig. 4D), TISIDB (Fig. 4E) and GEPIA (Fig. 4F) databases. Thus, CLDN18 was identified as a risk factor of HCC, and selected for subsequent analysis.

### Mutated genes and co-expressed genes associated with CLDN18 in HCC

**Fig. 5:**
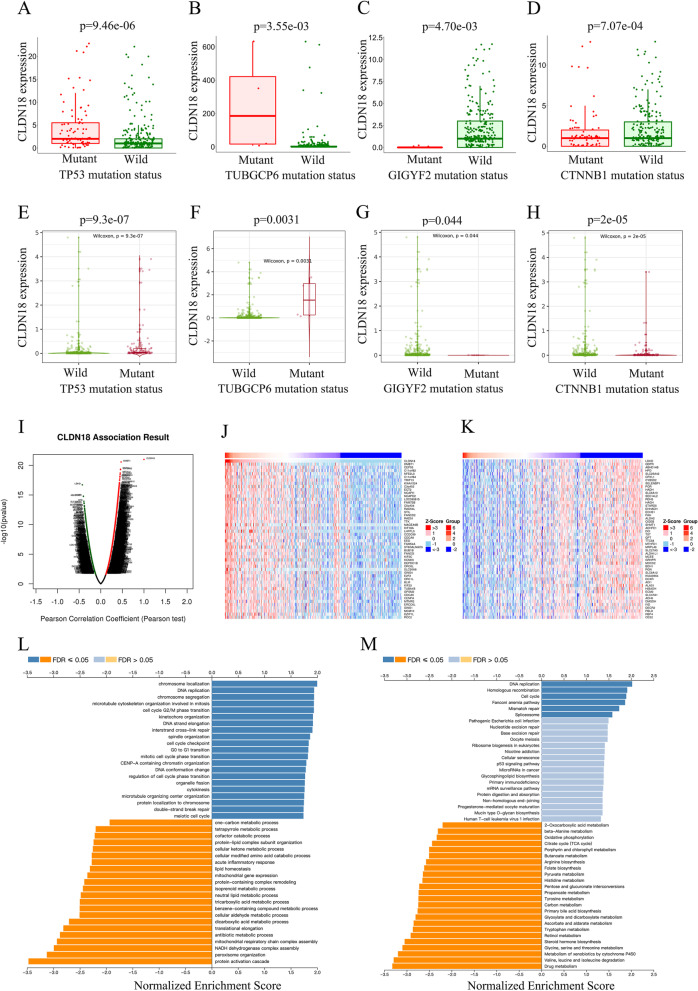
Functional annotation of genes co-expressed with CLDN18. **A**-**H** Correlation of TP53, CTNNB1, TUBGCP6 and GIGYF2 mutation status with CLDN18 in HCC (muTarget TIMER2.0). **I**  Correlation between DEGs and CLDN18. **J**, **K** Heat maps showing the top 50 genes positively (red) and negatively (blue) correlated with CLDN18 in HCC. **L** Biological functions of GO annotations. **M** KEGG pathways

The somatic mutations associated with CLDN18 in HCC were identified using the muTarget platform with *P* < 0.01 and 0.714 ＜ FC ＜1.4 as screening criteria. CLDN18 was significantly associated with mutated TP53, CTNNB1, TUBGCP6 and GIGYF2. CLDN18 was overexpressed in 28.49% of the TP53 mutant (*P* = 9.46E-06) and 1.12% of TUBGCP6 mutant HCC tissues compared to the wild-type tumors (Fig. 5A, B). In addition, CLDN18 was expressed at lower levels in samples harboring somatic mutations in CTNNB1 (mutation rate 24.3%) and GIGYF2 (1.4%) (Fig. 5C, D). This finding was verified in the TIMER2.0 database (Fig. 5E, F, G and H). To further explore the biological characteristics of CLDN18 in HCC, we analyzed the co-expressed genes (*P* < 0.05 and FDR < 0.01) using LinkedOmics database [17]. As shown in Fig. 5I, 6030 genes were positively correlated with CLDN18 (bright red dots) and 1384 genes were negatively correlated (dark green dots). The heatmaps of the top 50 positively and negatively correlated genes are shown in Fig. 5J and K. There was a strong positive correlation between CLDN18 and DMBT1 (positive rank#1, *r* = 0.471, *p* = 2.70E-21), CEP55 (*r* = 0.457, *p* = 4.50E-20) and C11orf82 (*r* = 0.457, *p* = 5.32E-20). Gene Set Enrichment Analysis (GSEA) further indicated that the genes co-expressed with CLDN18 are mainly involved in chromosome localization, DNA replication, chromosome segregation, inter-strand cross-link repair, microtubule cytoskeleton, cell cycle G2/M phase transition and other biological processes, while the biosynthetic process of cellular modified amino acid, protein-liquid complex subunit organization, acute biological processes such as inflammatory response and cellular ketone metabolic process were inhibited (Fig. 5L). KEGG pathway analysis showed that CLDN18 co-expressed genes were mainly enriched in DNA replication, homologous recombination, cell cycle and Fanconi anemia pathways (Fig. 5M) [18].

### CLDN18 is associated with immune infiltration in HCC

**Fig. 6:**
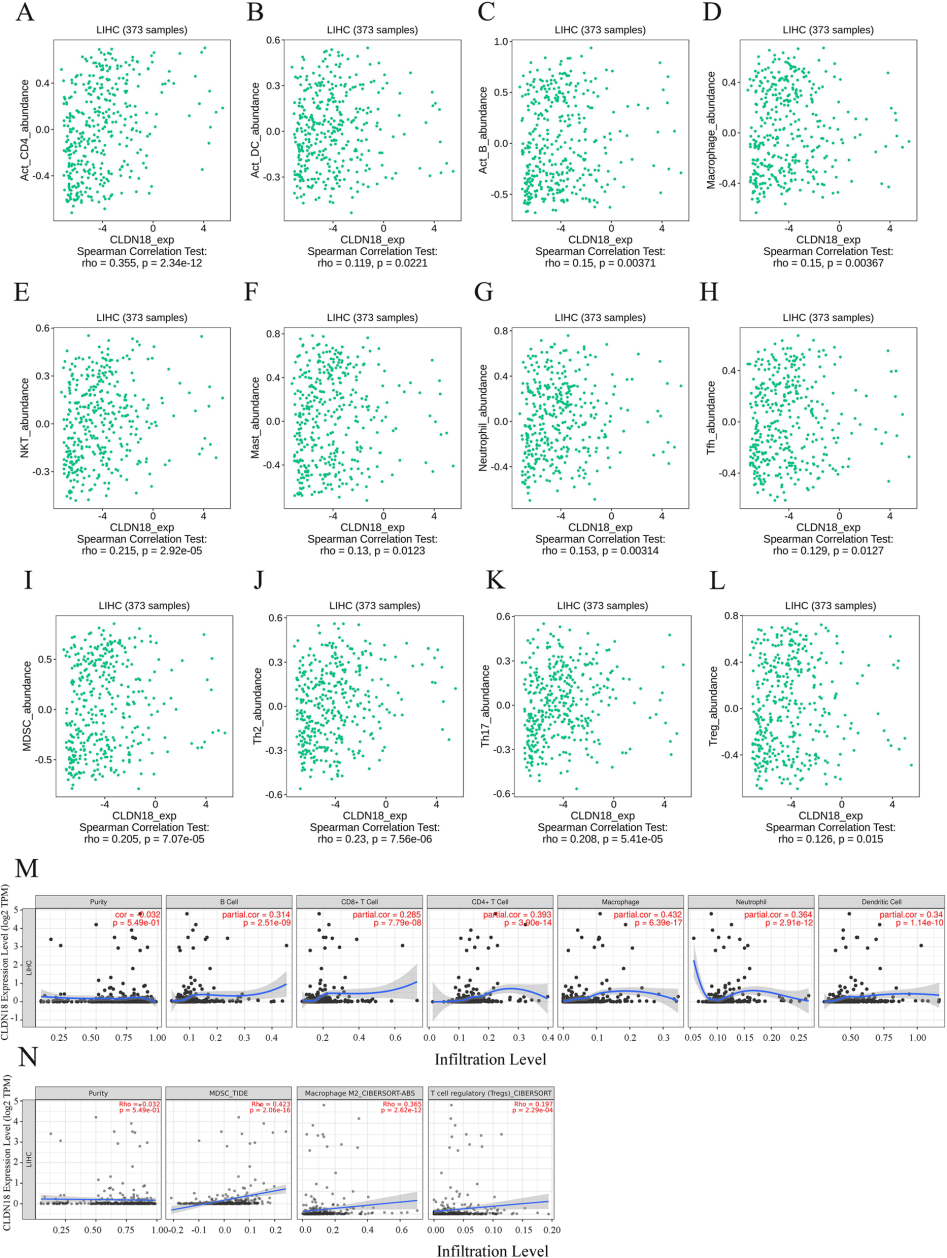
CLDN18 is associated with immune infiltration in HCC. **A**-**L** Correlation between CLDN18 and the abundance of infiltrating immune cells using TISIDB data. The immune cells include activated CD4 + T cells, activated B cells, macrophages, natural killer cells, mast cell, neutrophils, T follicular helper cells, T helper cell 2, T helper cell 17, regulatory T cells and myeloid-derived suppressor cells. **M** Association between CLDN18 and the infiltration levels of B cells, CD4 + T cells, CD8 + T cells, macrophages, neutrophils and dendritic cells in TIMER database. **N** CLDN18 was correlated with MDSCs, M2 macrophages and regulatory T cells in TIMER2.0 database

**Fig. 7:**
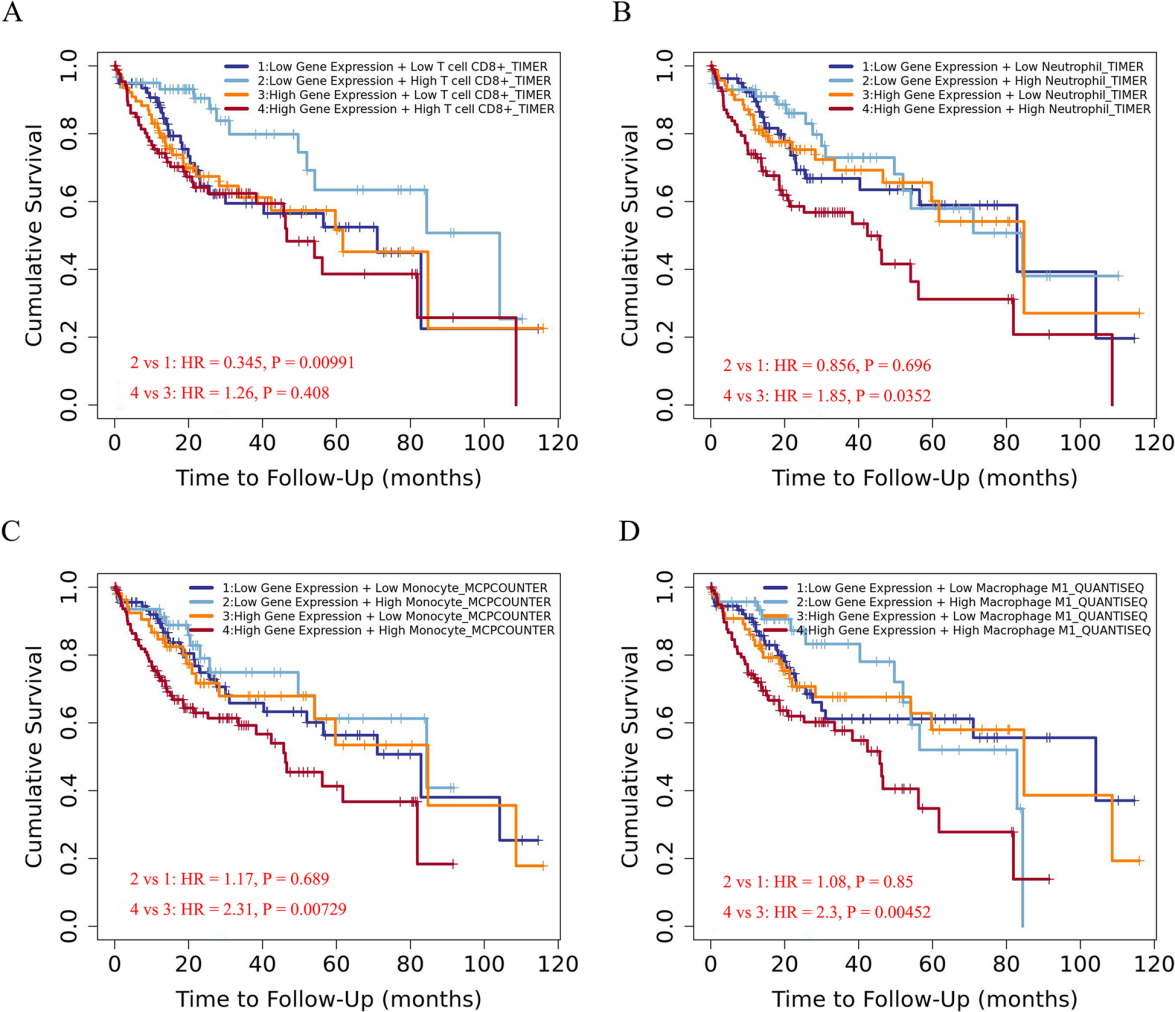
CLAN18 and the abundance of tumor immune cell infiltration affect the prognosis of HCC patients. **A** Kaplan–Meier curves of CLDN18/CD8 + T cells high or low HCC patients in the TIMER2.0 database. **B** Kaplan–Meier curves of CLDN18/neutrophils cells high or low HCC patients in the TIMER2.0 database. **C** Kaplan–Meier curves of CLDN18/ monocyte high or low HCC patients in the TIMER2.0 database. **D** Kaplan–Meier curves of CLDN18/ M1 macrophage high or low HCC patients in the TIMER2.0 database

The tumor immune microenvironment is a key factor driving tumor occurrence, progression, and metastasis. Through the TISIDB database, we found that CLDN18 is associated with activated CD4 + T cells (RHO = 0.355, *P* = 2.34E-12), activated dendritic cells (RHO = 0.119, *P* = 0.0221), activated B cells (RHO = 0.15, *P* = 0.00371), Mast cell (rho = 0.13, *P* = 0.0123), macrophages (rho = 0.15, *P* = 0.00367), natural killer cells (RHO = 0.251, *P* = 2.92E-05), neutrophils (RHO = 0.153, *P* = 0.00314) and T follicular helper cells (Fig. 6A-H), and positively correlated with the infiltration of inhibitory immune cells such as T helper cell 2 (RHO = 0.23, *P* = 7.56E-06), T helper cell 17 (RHO = 0.208, *P* = 5.41E-05), regulatory T cells (RHO = 0.126, *P* = 0.015) and myeloid-derived suppressor cells (RHO = 0.205, *P* = 7.07E-05) (Fig. 6I-L). The correlation between CLDN18 and immune cell infiltration was verified in the TIMER database (Fig. 6M, N). By applying the Multivariable Cox Proportional hazard model, we found that high CD8 + T cell infiltration levels predicted better survival in HCC with low CLDN18 expression (*P* = 0.0091, HR = 0.345), whereas high CLDN18 expression combined with infiltration of neutrophils (*P* = 0.0352, HR = 1.85), monocytes (*P* = 0.00729, HR = 2.31) and M1 macrophages (*P* = 0.00452, HR = 2.3) predicted poor prognosis (Fig. 7A-D).

### CLDN18 is associated with markers of inhibitory immune cells and T cells

**Fig. 8:**
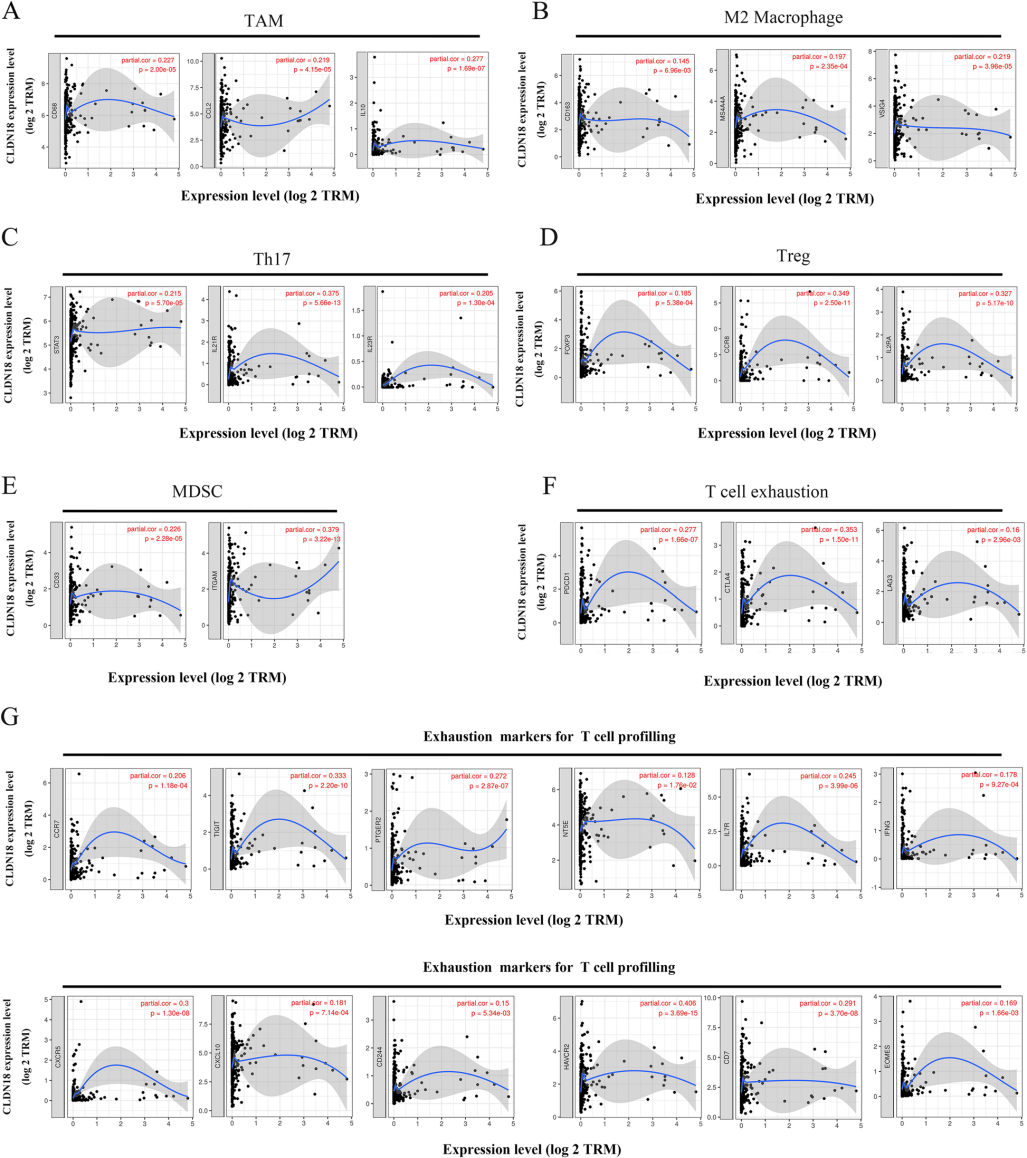
CLDN18 is associated with the markers of inhibitory immune cells and T cells in HCC. **A**-**F** Association of CLDN18 with CD68, CCL2 and IL10 of TAMs (tumor-associated macrophages), CD163, MS4A4A and VSIG4 of M2 macrophages, STAT3, IL21R and IL23R of T helper cell 17, FOXP3, CCR8 and IL2RA of regulatory T cells, CD33 and ITGAM of MDSCs (myeloid-derived suppressor cells), and PDCD1, CTLA4 and LAG3 of exhausted T cells. **G** Correlation of CLDN18 with exhaustion markers for T cells, including CCR7, TIGIT, PTGER2, NT5E, IL7R, IFNG, CXCR5, CXCL10, CD244, HAVCR2, CD7 and EOMES in HCC in the TIMER database

Immune escape of HCC tumors is driven by the infiltration and proliferation of inhibitory immune cells in the TME. As shown in Fig. 8A-F, CLDN18 was significantly associated with several biomarkers of inhibitory immune cells such as tumor-associated macrophages (TAMs; CD68, CCL2 and IL10, *p* < 0.0001), M2 macrophages (CD163, MS4A4A and VSIG4, *p* < 0.01), T helper cells 17 (STAT3, IL21R and IL23R, *p* < 0.0001), regulatory T cells (FOXP3, CCR8 and IL2RA, *p* < 0.0001), myeloid-derived suppressor cells (CD33 and ITGAM, *p* < 0.0001) and exhausted T cells (PDCD1, CTLA4 and LAG3, *p* < 0.01). T cells are key factors in the adaptive immune response, and their function, persistence and longevity determine the efficacy of immunotherapies and patient prognosis. To this end, we analyzed the correlation between CLDN18 and multiple T cell markers. CLDN18 correlated significantly with the markers T cell depletion, including CCR7 (COR = 0.206, *P* = 1.18E-04), TIGIT (COR = 0.333, *P* = 2.20E-10), PTGER2 (COR = 0.272, *P* = 2.87E-07), NT5E (COR = 0.128, *P* = 1.76E-02), IL7R (COR = 0.245, *P* = 3.99E-06), IFNG (COR = 0.178, *P* = 9.27E-04), CXCR5 (COR = 0.3, *P* = 1.3E-08), CXCL10 (COR = 0.181, *P* = 7.14E-04) and CD244 (COR = 0.15, *P* = 5.34E-03) (Fig. 8G). In addition, CLDN18 was significantly associated with markers of activated CD8 + T cells and CD4 + T cells after adjusting for tumor purity. As shown in Table 1, the CD8 + T cell markers included MPZL1 (Positive rank # 1COR = 0.472, *P* = 1.66E-20), CSE1L (COR = 0.422, *P* = 2.57E-16), CD37 (COR = 0.38, *P* = 2.57E-13), AHSA1 (COR = 0.324, *P* = 6.91E-10), CD3D (COR = 0.321, *P* = 9.91E-10) and IL2RB (COR = 0.307, *P* = 5.66E-09), and that of CD4 + T cells were PRC1 (Positive rank # 1COR = 0.534, *P* = 8.28E-27), NUF2 (COR = 0.524, *P* = 1.06E-25), KIF11 (COR = 0.524), EXO1 (COR = 0.508, *P* = 4.96E-24), RTKN2 (COR = 0.491, *P* = 2.30E-22) and CCNB1 (COR = 0.485, *P* = 8.49E-22).

**Table 1:** Correlation between CLDN18 and markers of activated T cells

Activated CD8 T cell	None		Purity		Activated CD4 T cell	None		Purity	
	**Cor**	***P***	**Cor**	**P**		**Cor**	**P**	**Cor**	**P**
ADRM1	0.235	***	0.221	***	AIM2	0.258	***	0.263	***
AHSA1	0.295	***	0.324	***	BIRC3	0.357	***	0.368	***
C1GALT1C1	0.205	***	0.221	***	BRIP1	0.309	***	0.345	***
CCT6B	-0.176	***	-0.032	0.549	CCL20	0.356	***	0.339	***
CD37	0.346	***	0.38	***	CCL4	0.181	***	0.178	***
CD3D	0.311	***	0.321	***	CCL5	0.207	***	0.2	***
CD3E	0.278	***	0.297	***	CCNB1	0.477	***	0.485	***
CD3G	0.27	***	0.268	***	CCR7	0.197	***	0.206	***
CD69	0.229	***	0.24	***	DUSP2	0.283	***	0.267	***
CD8A	0.224	***	0.218	***	ESCO2	0.462	***	0.464	***
CETN3	0.294	***	0.278	***	ETS1	0.222	***	0.235	***
CSE1L	0.389	***	0.422	***	EXO1	0.493	***	0.508	***
GEMIN6	0.22	***	0.244	***	EXOC6	0.37	***	0.375	***
GNLY	0.089	0.0874	0.062	0.251	IARS	0.385	***	0.392	***
GPT2	-0.284	***	-0.268	***	ITK	0.25	***	0.263	***
GZMA	0.155	**	0.14	**	KIF11	0.507	***	0.524	***
GZMH	0.048	0.358	0.027	0.611	KNTC1	0483	***	0.497	***
GZMK	0.142	**	0.135	0.0123	NUF2	0.505	***	0.524	***
IL2RB	0.291	***	0.307	***	PRC1	0.509	***	0.534	***
LCK	0.269	***	0.28	***	PSAT1	0.149	**	0.157	**
MPZL1	0.468	***	0.472	***	RGS1	0.396	***	0.417	***
NKG7	0.049	0.344	0.016	0.773	RTKN2	0.476	***	0.491	***
PIK3IP1	0.264	***	0.262	***	SAMSN1	0.31	***	0.347	***
PTRH2	0.23	***	0.239	***	SELL	0.14	**	0.142	**
TIMM13	0.132	0.0107	0.119	0.0266	TRAT1	0.229	**	0.24	**
ZAP70	0.219	***	0.22	***					

Cor, *P* value of Spearman’s correlation; None, correlation without adjustment. Purity, correlation adjusted by purity. * *p* < 0.05,** *p* < 0.01,*** *p* < 0.001

## Discussion

The liver has a unique immunological structure compared to other major organs. A large number of immune cells patrol the liver, while maintaining a certain amount of immune tolerance against the chronic antigenic load of gastrointestinal origin [2, 19]. The bidirectional effects of immunotolerance and immune-activation create challenges for anti-HCC immunotherapy. Therefore, there is an urgent need to identify biomarkers for evaluating prognosis and monitoring treatment [20, 21]. Non-invasive liquid biopsies are increasingly being used for early cancer screening through the detection of mutated genes in circulating tumor cells (CTCs) or quantification of CTC DNA (ctDNA) levels [22, 23].

Tumor growth significantly affects the function and components of the immune system [24]. For instance, tumor cells can hide from immune surveillance and induce immune escape by down-regulating major histocompatibility complex (MHC), and secreting immunosuppressive cytokines, exosomes, microvesicles, etc [20, 25, 26]. The function and abundance of immune effector cells and antigen presenting cells (APCs) in the TME are key determinants of tumor progression and prognosis [21, 27, 28]. PBMCs are an abundant and easily accessible source of immune cells and genes, and are therefore an effective substitute for tumor tissue biopsies [29]. The aim of this study was to identify the circulating prognostic biomarkers of HCC using PBMCs harvested from patients.

Based on RNA sequencing of the PBMCs from HCC patients, we identified METTL7B, BCAM, CLDN18, DEFA1B, SOCS3, HBG2, GSTM1, RNASE3, ITGA9 and S100P as potential markers associated with poor prognosis, of which METTL7B, ITGA9, SOCS3 and RNASE3 were significantly upregulated in BCLC stage D patients. Furthermore, CLDN18 was significantly overexpressed in HCC tumor tissues compared to the adjacent non-tumor tissues, which was consistent with PBMC sequencing results and also validated with the immunohistochemical data from human protein profiles. CLDN18 also correlated positively with HCC stage and grade, and high expression levels of CLDN18 predicted shorter overall survival. Together, these data suggested that CLDN18 is a potential biomarker for predicting HCC prognosis.

Claudin 18 (CLDN18) is a transmembrane protein of the Claudin family, and CLDN18 mRNA is normally expressed in the lungs and stomach. Previous studies have shown that CLDN18 is significantly downregulated in gastric cancer tissues, but is elevated in patients with bone metastasis [30]. High expression of CLDN18.2 has been associated with diffuse histological variation of gastric adenocarcinoma, and its decrease with the progression of gastric adenocarcinoma [31]. CLDN18.1 inhibits IGF-1R/AKT and YAP/TAZ/AKT signaling pathways in lung adenocarcinoma cells, which correlates with favorable prognosis [32]. In a previous study, we found that CLDN18 is highly expressed in liver cancer tissues as well as in the PBMCs of the patients. In addition, CLDN18 levels were higher in patients with TP53 and TUBGCP6 mutations compared to their wild-type counterparts, and portended poor prognosis. Enrichment analysis showed that CLDN18 is mainly involved in chromosome localization, DNA replication, chromosome segregation, inter-strand cross-link repair, microtubule cytoskeleton, cell cycle G2/M phase transition and other biological processes. Moreover, CLDN18 participates in the inhibition of modified amino acid biosynthesis, protein-liquid complex subunit organization, acute inflammatory response, and ketone metabolism. KEGG pathway analysis further showed that CLDN18 and its co-expressed genes are mainly enriched in DNA replication, homologous recombination, cell cycle and Fanconi anemia pathways.

Immune invasion is a predictor of tumor prognosis, and CLDN18 is correlated with the extent of immune cell infiltration in HCC tumors. We found a positive correlation between CLDN18 expression and the infiltration of CD4 + T cells, CD8 + T cells DCs, B cells, macrophages, NK cells, neutrophils, as well as immunosuppressive subsets such as Th2 cells, Th17 cells, Tregs, TAMs and MDSCs. In addition, low expression of CLDN18 and high infiltration of CD8 + T cells predicted a better prognosis, while high expression of CLDN18 along with high infiltration of neutrophils, monocytes and M1 macrophages was associated with shorter survival. CLDN18 also correlated with the markers of inhibitory immune cells such as TAMs (CD68, CCL2 and IL10), M2 macrophages (CD163, MS4A4A and VSIG4), Th17 cells (STAT3, IL21R and IL23R), Tregs (FOXP3, CCR8 and IL2RA) and MDSCs (CD33 and ITGAM).

T cells are the main effectors of the adaptive immune response [33]. The differentiation of T cells is disrupted in the TME, and the ensuing epigenetic and metabolic changes lead to a gradual loss of effector function and proliferation ability, eventually resulting in tumor immune escape [26, 34]. Overexpression of immune checkpoint molecules is an important marker of T cell depletion. The inhibitory signals relayed by immune checkpoints prevent excessive immune responses, whereas overexpression of checkpoint molecules in the TME inhibits anti-tumor responses and aids in immune escape [35, 36]. We found that checkpoint molecules such as PD-1, CTLA4 and LAG3, as well as markers of exhausted T cells such as CCR7, TIGIT, PTGER2, NT5E, IL7R, IFNG, CXCR5, CXCL10 and CD244 were associated with CLDN18 in HCC. In addition, CLDN18 also correlated significantly with markers of activated CD8 + T cells such as MPZL1, CSE1L, CD37, AHSA1, CD3D and IL2RB, after adjusting for tumor purity. For activated CD8 + T cells, CLDN18 has a synergistic relationship with PRC1, NUF2, KIF11, EXO1, RTKN2, CCNB1 and other marker genes in HCC. Taken together, CLDN18 is likely involved in immune cell infiltration in HCC tumors.

There are several limitations in our study that ought to be considered. First, the clinical samples were limited, and more sequencing data is required for validation. Secondly, the immunomodulatory effects of CLDN18 on HCC need to be further verified by in vitro and in vivo experiments. Moreover, the mechanism underlying immune regulation needs to be explored. Nevertheless, we identified CLDN18 overexpression as an independent factor of poor prognosis for HCC, which provides new insights into the pathological mechanism of HCC progression, especially in the context of tumor immune environment.

## Conclusions

CLDN18 was significantly upregulated in the PBMCs and tumor tissues of HCC patients, and correlated with the abundance of immune cell infiltration. It is a potential prognostic biomarker and therapeutic target in HCC.

## Supplementary Information

**Additional file 1.**

## Data Availability

The datasets generated and/or evaluated during the current study are available at NCBI project PRJNA909469 (https://www.ncbi.nlm.nih.gov/bioproject/PRJNA909469).
