# Supplementary figures and images for: Identification of immune-related target and prognostic biomarkers in PBMC of hepatocellular carcinoma
Source: BMC Gastroenterol. 2023 Jul 12;23:234. doi: 10.1186/s12876-023-02843-y (PMC10337054; doi:10.1186/s12876-023-02843-y)

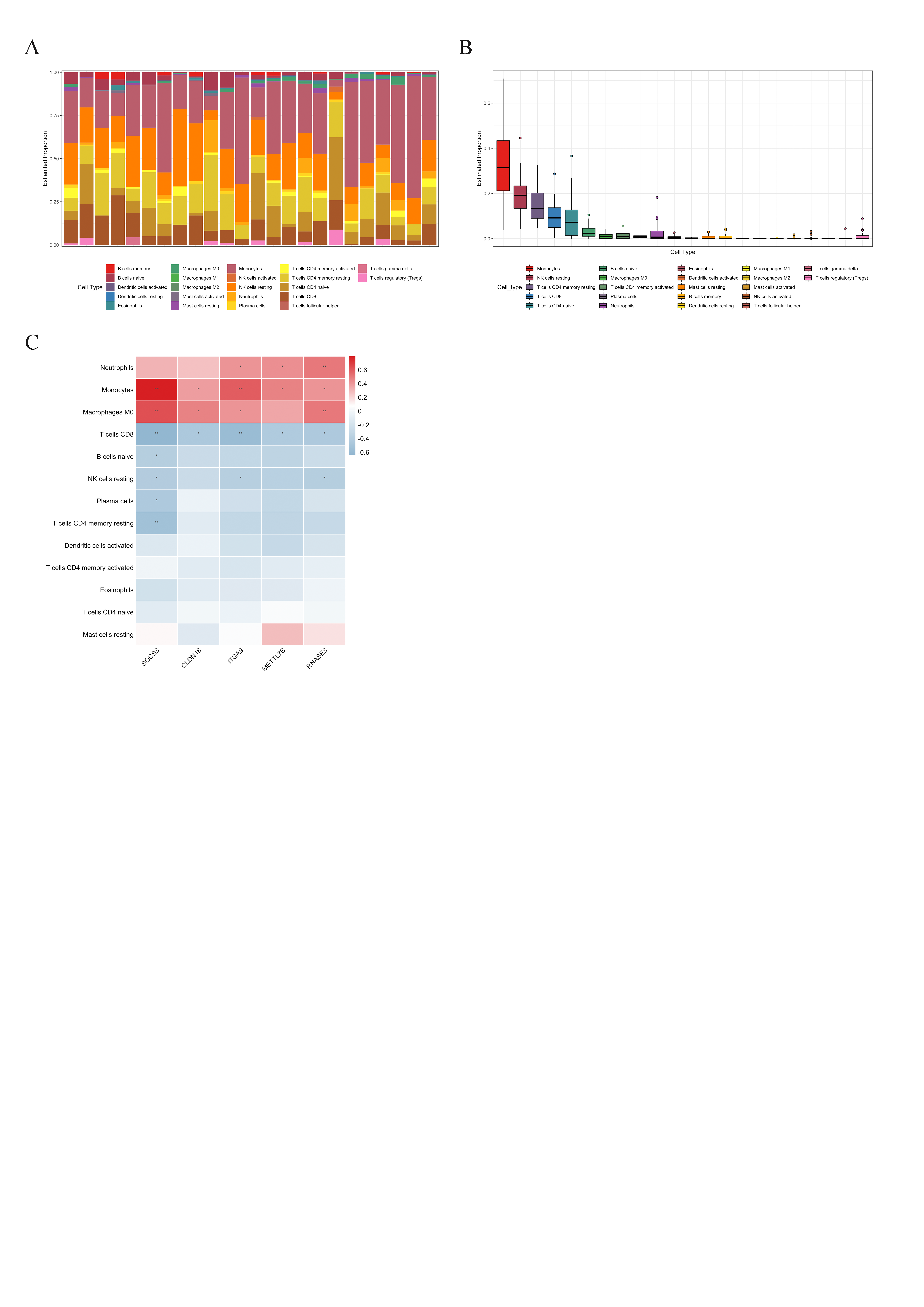

Supplement: Supplementary file 1 — Additional file 1. [file 12876_2023_2843_MOESM1_ESM.tif]
